# Causal association between rheumatoid arthritis and celiac disease: A bidirectional two-sample mendelian randomization study

**DOI:** 10.3389/fgene.2022.976579

**Published:** 2022-10-18

**Authors:** Lijiangshan Hua, Shate Xiang, Rixiang Xu, Xiao Xu, Ting Liu, Yanan Shi, Lingyun Wu, Rongyun Wang, Qiuhua Sun

**Affiliations:** ^1^ School of Nursing, Zhejiang Chinese Medical University, Hangzhou, Zhejiang, China; ^2^ School of Basic Medical Sciences, Zhejiang Chinese Medical University, Hangzhou, Zhejiang, China; ^3^ School of Humanities and Management, Zhejiang Chinese Medical University, Hangzhou, Zhejiang, China

**Keywords:** mendelian randomization, rheumatoid arthritis, celiac disease, bidirectional, causality

## Abstract

**Objectives:** Rheumatoid Arthritis (RA) has been associated with Celiac Disease (CD) in previous observational epidemiological studies. However, evidence for this association is limited and inconsistent, and it remains uncertain whether the association is causal or due to confounding or reverse causality. This study aimed to assess the bidirectional causal relationship between RA and CD.

**Methods:** In this two-sample Mendelian randomization (MR) study, instrumental variables (IVs) for RA were derived from a genome-wide association studies (GWAS) meta-analysis including 58,284 subjects. Summary statistics for CD originated from a GWAS meta-analysis with 15,283 subjects. The inverse-variance weighted (IVW) method was used as the primary analysis. Four complementary methods were applied, including the weighted-median, weighted mode, MR pleiotropy residual sum and outlier (MR-PRESSO) test and MR-Egger regression, to strengthen the effect estimates.

**Results:** Positive causal effects of genetically increased RA risk on CD were derived [IVW odds ratio (OR): 1.46, 95% confidence interval (CI): 1.19–1.79, *p =* 3.21E-04]. The results of reverse MR analysis demonstrated no significant causal effect of CD on RA (IVW OR: 1.05, 95% CI: 0.91–1.21, *p =* 0.499). According to the sensitivity analysis, horizontal pleiotropy was unlikely to distort the causal estimates.

**Conclusion:** This study reveals a causality of RA on CD but not CD on RA among patients of European descent. This outcome suggests that the features and indicators of CD should regularly be assessed for RA patients.

## Introduction

Rheumatoid arthritis (RA) is a multi-systemic inflammatory autoimmune disease characterized by synovitis and joint damage, with a prevalence of 0.5%–1% (Smolen et al., 2016). Numerous studies demonstrated that RA causes a heavy burden on both individuals and society (Cutolo et al., 2014; Safiri et al., 2019; Hsieh et al., 2020). Celiac disease (CD) is an autoimmune disorder that occurs in genetically predisposed individuals who develop an immune reaction to gluten, with a worldwide prevalence of 1–2% (Gnodi et al., 2022). It is often accompanied by either, or both, intestinal and non-intestinal symptoms, such as diarrhea, steatorrhea, constipation, weight loss, anemia, hypo-proteinemia, and osteoporosis (Rej and Sanders, 2021). Genetic and some environmental factors, such as alteration of the gut microbiome and inflammation are believed responsible for the development of RA and CD (Smolen et al., 2016; Lebwohl et al., 2018). Genetically, the human leukocyte antigen (HLA) risk alleles play an essential role in the susceptibility of RA and CD. Individuals carrying HLA-DR shared epitope alleles have an increased risk of developing RA, whereas those carrying HLA-DQ2.5 and/or HLA-DQ8 alleles are more likely to develop celiac disease (Koning et al., 2015).

The association between RA and CD has recently received much attention (Lerner and Matthias, 2015). It has been estimated that the prevalence rate of CD in RA patients is approximately 3%, which is triple the healthy population (Elhami et al., 2018). Results from several cross-sectional and retrospective studies highlight that CD is associated with a high frequency of rheumatoid factor-IgA (RF-IgA), implying the prevalence of RA in CD patients might be higher than in healthy controls (Fayyaz et al., 2019; Ghozzi et al., 2022). Moreover, a recent epidemiological study clarifies that children with one multiple chronic inflammatory diseases (CIDs) affected parent are at a higher risk of developing the same CIDs as their parents as well as other specific CIDs reliant on the parents’ CIDs (Andersen et al., 2021). Given that, children of patients comorbid with both RA and CD are considered at an increased risk for developing RA and CD in the future compared to children with no diseased parents (Andersen et al., 2021). In addition, patients accompanied by both RA and CD have a higher risk of osteoporosis and fractures, which would largely decrease the life quality and increase the risk of mortality of patients (Choi et al., 2018; Ganji et al., 2019).

Even though the exact mechanisms of the relationship between RA and CD observed in the epidemiological and observational studies are not fully understood, the gut-joint axis hypothesis was proposed as an indispensable explanation of the pathogenic link (Lerner and Matthias, 2015). Abnormal intestinal barrier permeability occurs not only in patients with CD (Lerner and Matthias, 2015) but also in RA patients (Zaiss et al., 2021). The primary mechanism of barrier disruption in the gut is potentially *via* increased zonulin production, an essential regulator of the integrity of the tight junctions in the intestinal epithelium (Fasano, 2020). Notably, identified triggers for zonulin release from intestinal epithelial cells include gluten (Drago et al., 2006), a protein that causes CD, and dysbiotic microbiota (El Asmar et al., 2002; Ciccia et al., 2017). Furthermore, autoantibodies related to RA could be generated within the inflamed intestine. Pro-inflammatory immune cells primed in intestinal tissues could traffic to the joints and systemic sites, exacerbating inflammation in genetically susceptible individuals and contributing to RA and CD occurrence (Teng et al., 2016; Zaiss et al., 2021). In addition, a moderate inflammation of the small bowel mucosa has been reported with an increased number of intraepithelial lymphocytes (IELs) in patients with RA (Molberg and Sollid, 2006). IELs have been observed to migrate from joints to the gut mucosa and *vice versa*. Notably, CD4^+^ T lymphocytes detected in synovial fluid of RA patients have been demonstrated to express NKG2D, one of the NK-cell family receptors and a typical IEL marker of CD patients. However, observational studies might be confounded by potential confounding factors and reverse causation. Whether the observed relationships between RA and CD reflect causality requires more investigation.

Mendelian randomization (MR) is used to determine any association between risk factors and disease outcomes by employing genetic variations as instrumental variables (IVs), per the law of independent assortment, where genetic variants are allocated randomly at conception (Davey Smith and Hemani, 2014; Yarmolinsky et al., 2018; Mukamal et al., 2020). This statistical approach avoids confusion and the bias associated with reverse causation since genotypes precede the disease process and are usually unaffected by postnatal lifestyle or environmental influences (Ebrahim and Davey Smith, 2008; Lawlor et al., 2008). Based on the current genetic databank, genetic variants controlling RA could be utilized as IVs to investigate the effect of RA on the risk of developing CD, thus removing confounding variables from the data.

No MR analysis has been reported investigating a possible causal relationship between RA and CD. Investigating the causal relationship between these two diseases is of great significance since it will consolidate existing knowledge of RA and CD pathogeneses and improve treatments. This study is the first MR analysis to examine the potential causal relationships of genetically predicted RA with the risk for CD. We also undertook reverse MR to investigate the causal effect of CD on RA.

## Materials and methods

### Ethics/consent statement

No further ethical approval or participation consent was required, as this study drew on published articles and public databases.

**FIGURE 1:**
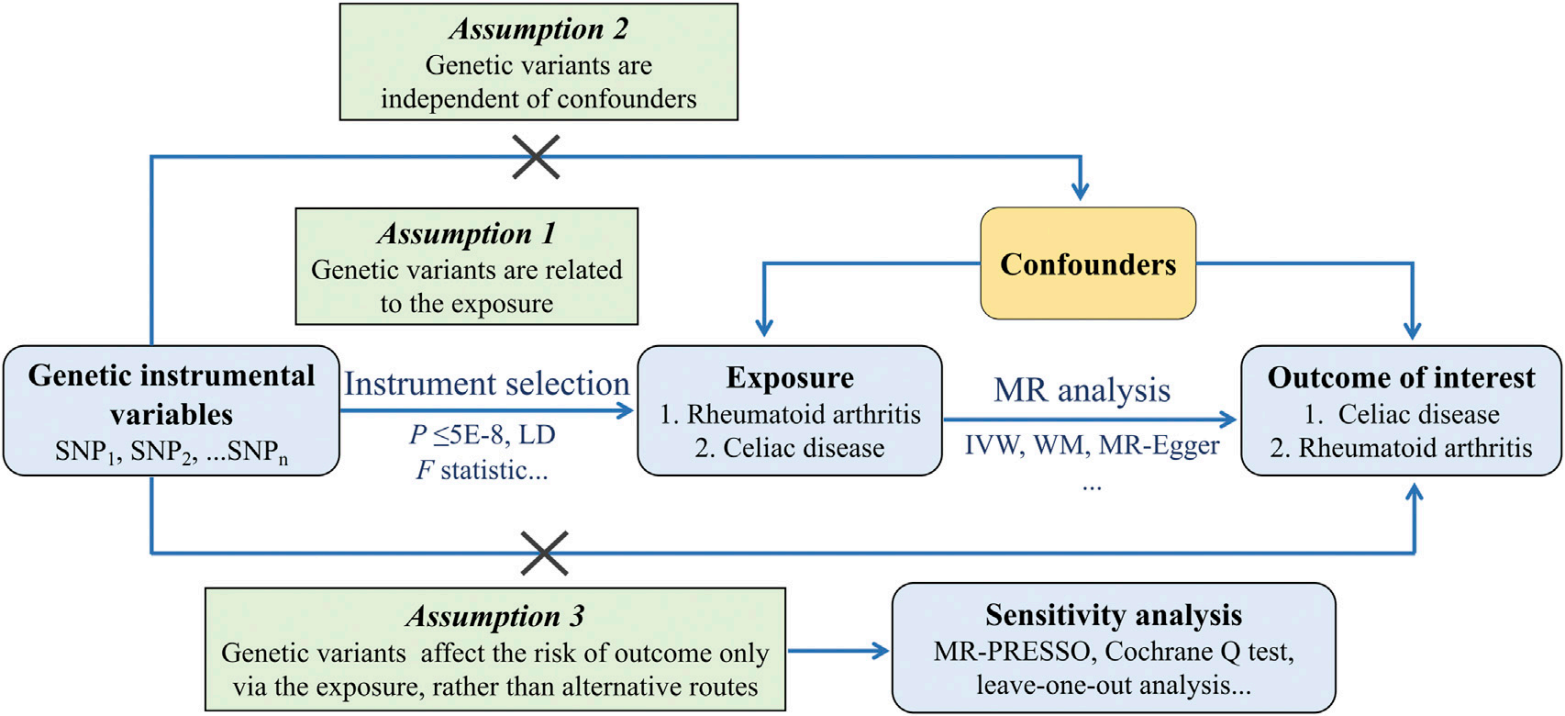
Schematics for the bidirectional MR design. Abbreviation: MR, Mendelian randomization; SNP, single nucleotide polymorphism; LD, linkage disequilibrium; IVW, Inverse Variance Weighted; WM, Weighted Median; PRESSO, Pleiotropy REsidual Sum and Outlier.

The framework of the two-sample MR study is shown in Figure 1. Genetic variations were used to investigate the causal relationship of RA on CD and the reverse causation separately. To obtain reliable results, selected IVs must meet three essential assumptions: 1) the IVs are strongly related to the exposure, 2) the IVs have no relationship to any confounders affecting both exposure and outcome, and 3) the IVs influence outcome only through the exposure. As for each inference direction, the MR analysis includes three key procedures: extracting single-nucleotide polymorphisms (SNPs) associated with interested exposure as IVs, performing primary MR analysis, and for significant associations, a series of sensitivity analysis procedures were undertaken.

### Data source

In this MR study, a crucial step was to choose appropriate genetic variants from the publicly available genome-wide association studies (GWAS) database. The selected SNPs as IVs were chosen for exposures and outcomes from the IEU GWAS database (https://gwas.mrcieu.ac.uk/datasets/).

Summary statistics for RA originated from a large-scale GWAS meta-analysis involving 58,284 subjects of European ancestry (14,361 RA cases and 43,923 controls) (Okada et al., 2014). SNPs associated with CD were derived from a GWAS meta-analysis including 15,283 subjects of European ancestry (4,533 CD cases and 10,750 controls) (Dubois et al., 2010) (Supplementary Table S1).

Potentially, population stratification may introduce bias into MR analysis. Since the allele frequencies differ, a single SNP could be associated with ancestry, whereas it may be related to disease risk. SNPs and their corresponding summary statistics in the MR analysis were restricted to European descent for the exposures and outcomes to mitigate this bias.

### Selection of instrumental variables

A series of quality control steps were performed to select eligible SNPs. Firstly, SNPs associated with exposures were extracted with genome-wide significance (*P* < 5E–08), which were the potential IVs. Secondly, independent SNPs were selected *via* setting the linkage disequilibrium (LD) threshold for clumping to *r* ^2^ < 0.01, and the clumping window size was 5,000 kb. The independent SNPs could not have an overlap with the reported fourteen shared loci between RA and CD (Zhernakova et al., 2011). Moreover, if the *r* ^2^ of these independent SNPs and the fourteen shared loci were greater than 0.01, the independent SNP would also be excluded from the IVs. Thirdly, to satisfy the assumptions of eligible IVs, SNPs associated with traits of outcomes were excluded by manually searching in the PhenoScanner GWAS database (http://phenoscanner.medschl.cam.ac.uk). Fourthly, SNPs with a minor allele frequency (MAF) less than 0.01 were also eliminated. Finally, the effect alleles of genetic instruments were harmonized across the exposure and outcome GWAS.

The *F* statistics were calculated to assess the strength of the selected IVs. If the *F* statistic is much greater than 10 for the instrument-exposure association, the possibility of weak instrumental variable bias is slight (Pierce et al., 2011).

### Statistical analysis

This study applied multiple complementary methods, including the inverse variance weighted (IVW) method, the MR-Egger regression, the weighted median (WM) approach, and the weighted mode regression, to investigate the causal relationship between exposures and outcomes.

Specifically, the fixed-effects or random-effects IVW method was performed as the primary analysis of causal estimates, which would provide the most precise results when all the IVs were valid (Burgess et al., 2020). The WM approach uses the median MR estimate as the causative estimate (Bowden et al., 2016), and the MR Egger regression allows the intercept to indicate average pleiotropic bias (Bowden et al., 2015). These two methods are relatively robust to horizontal pleiotropy at the sacrifice of statistical power. Moreover, the weighted mode method could assess the causal association of the subset with the largest number of SNPs *via* clustering the SNPs into subsets resting on the resemblance of causal effects (Hartwig et al., 2017).

Additionally, the MR Pleiotropy RESidual Sum and Outlier (MR-PRESSO) test was applied to detect potential horizontal pleiotropy and correct it by removing outliers. The Cochrane Q test was used to evaluate heterogeneity between SNPs in the IVW method. When heterogeneity exists (*p* < 0.05), the random-effects IVW test was utilized to provide a more conservative yet robust estimate. At last, the leave-one-out analysis was performed to guarantee the reliability of the affiliation between the SNPs and exposures, evaluating whether any SNP was responsible for the significant results.

All the bidirectional MR analyses were undertaken using R (version 4.1.3) with the “*TwoSampleMR*” and the “*MRPRESSO*” packages.

## Results

### Effects of rheumatoid arthritis on celiac disease

After a series of approaches selecting eligible IVs and excluding potential pleiotropic SNPs, five SNPs strongly related to RA were identified as IVs in the MR analysis (Supplementary Table S2). These 5 SNPs explain 3% of the variance in RA across the population. The *F* statistic of these SNPs ranged from 210 to 528, indicating the instrument was sufficiently robust to eliminate the potential of null association due to instrument bias (Pierce et al., 2011).

**FIGURE 2:**
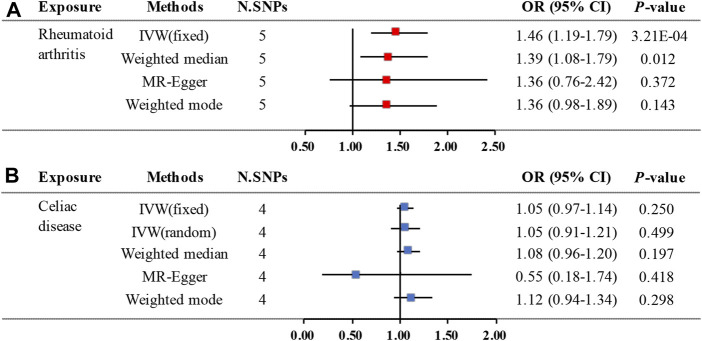
Two-sample MR estimates results of causal associations between genetically predicted RA and CD. **(A)** Causal estimates result for RA on CD. **(B)** Causal estimates result for CD on RA. Abbreviation: MR, Mendelian randomization; RA, rheumatoid arthritis; CD, celiac disease; N.SNPs is the number of SNPs being used as IVs; SNPs, single nucleotide polymorphisms; OR, odds ratio; CI, confidence interval; IVW, Inverse Variance Weighted.

**FIGURE 3:**
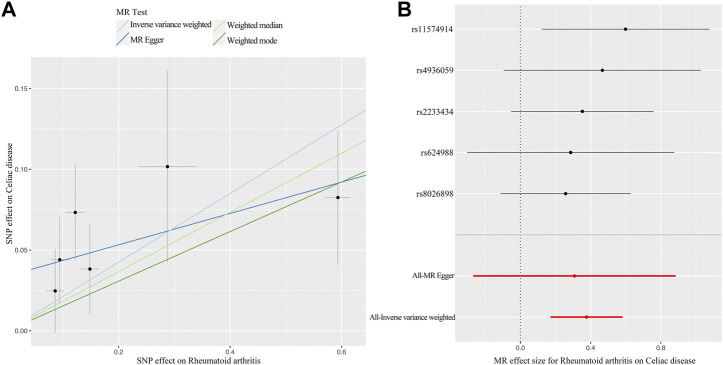
MR plots for the causal effect of RA on CD. **(A)** Scatter plot for the causal relationship of RA on CD. **(B)** Forest plot for the causal relationship of RA on CD. Abbreviation: MR, Mendelian randomization; RA, rheumatoid arthritis; CD, celiac disease; SNP, single nucleotide polymorphism.

The primary analysis indicated a significant causal relationship between an increased risk of RA and changes in CD risk (IVW OR: 1.46, 95% CI: 1.19–1.79, *p =* 3.21E-04) (Figure 2). The WM method yielded the same pattern of effects (OR: 1.39, 95% CI: 1.08–1.79, *p =* 0.012). Moreover, the MR-PRESSO test and the MR-Egger regression did not detect any horizontal pleiotropy among the instrumental SNPs (Table 1). No heterogeneity was observed in the Cochrane Q test (Table 1). The result of the leave-one-out analysis demonstrated that the risk estimate of genetically predicted RA on CD was remarkably stable after leaving out one SNP at a time (Supplementary Figure S1). The scatter plots and forest plots are presented in Figure 3.

**TABLE 1:** Heterogeneity and horizontal pleiotropy analyses between RA and CD.

Exposure	Outcome	MR-PRESSO global test		MR-Egger		IVW	
		RSSobs	*p*-value	Intercept	*p-*intercept	Q statistic	Q-pval
RA	CD	2.34	0.877	0.009	0.819	1.43	0.839
CD	RA	16.84	0.12	0.175	0.384	8.66	0.034

### Effects of celiac disease on rheumatoid arthritis

In the reverse MR analysis, four significant (*P* < 5E–08) and independent SNPs (*r* ^2^ < 0.01) were incorporated as IVs for CD and explained 6.9% of the phenotypic variation. All the *F* statistics are greater than 10 (ranging from 205 to 301), indicating no evidence of weak instrument bias (Supplementary Table S3).

**FIGURE 4:**
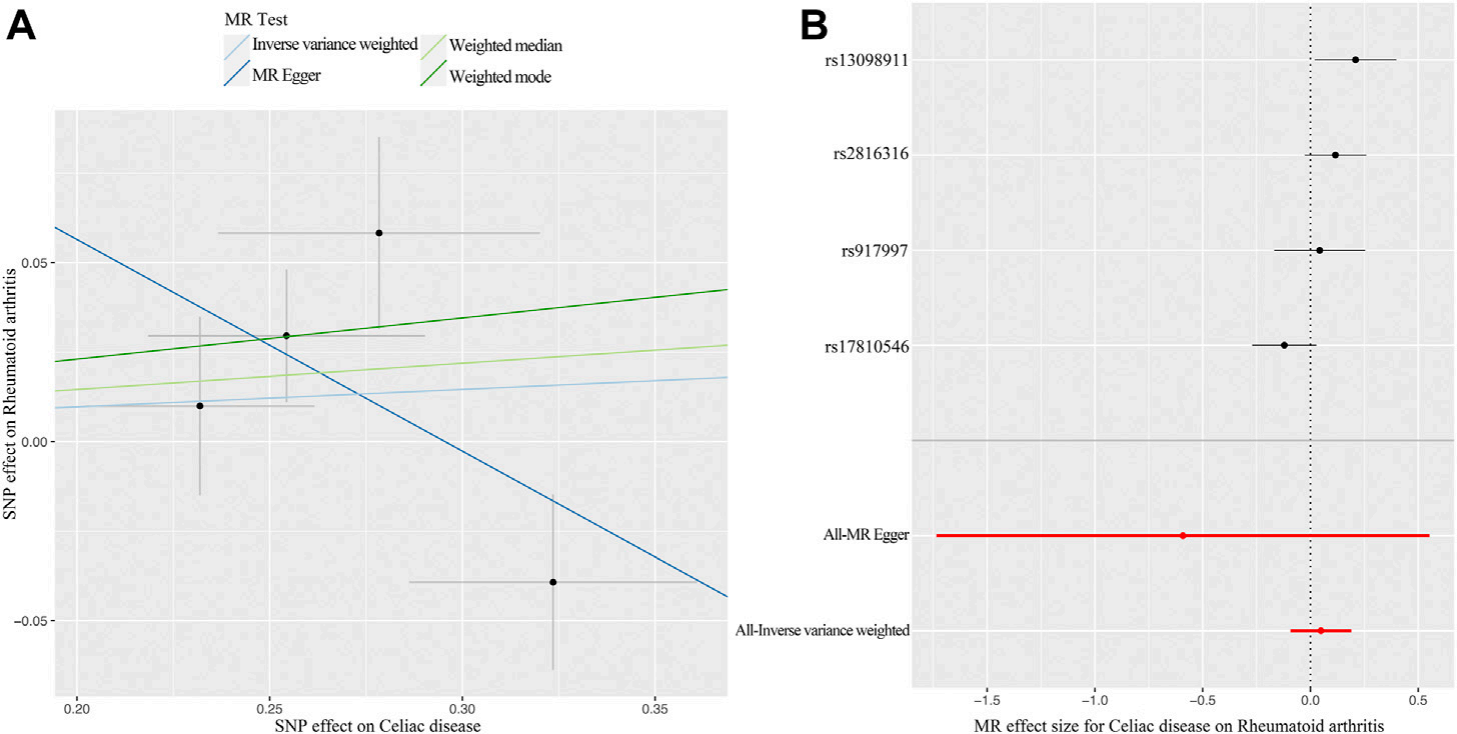
MR plots for the causal effect of CD on RA. **(A)** Scatter plot for the causal relationship of CD on RA. **(B)** Forest plot for the causal relationship of CD on RA. Abbreviation: MR, Mendelian randomization; CD, celiac disease; RA, rheumatoid arthritis; SNP, single nucleotide polymorphism.

The MR analysis demonstrated that genetic liability to RA is not significantly associated with CD diagnosis. To be specific, the corresponding effect estimate is 1.05 (95% CI: 0.97–1.14, *p =* 0.250) in the IVW (fixed effects) method and remained consistent in the WM method (OR: 1.08, 95% CI: 0.96–1.20, *p =* 0.197) (Figure 2). The MR-PRESSO test results indicate no outlier, and the MR-Egger intercept did not identify any pleiotropic SNPs. However, the Cochrane Q test evidences the existence of slight heterogeneity (*p =* 0.034) (Table 1). Then, the IVW method based on the multiplicative random effects was performed, indicating that the onset of RA was not causally associated with suffering from CD (OR: 1.05, 95% CI: 0.91–1.21, *p =* 0.499) (Figure 2). Finally, the leave-one-out analysis demonstrated that the observed relationship was not driven by a single SNP (Supplementary Figure S2). Scatter plots and forest plots are shown in Figure 4.

## Discussion

This study is the first MR analysis to investigate the bidirectional causal association between RA and CD, using large-scale GWAS data by conducting multiple MR approaches. The results suggest that genetically predicted RA is causally related to CD in individuals of European descent. Conversely, the current study did not observe evidence supporting that genetically predicted CD was associated with an increased risk of RA.

Previous observational studies have investigated the association between RA and CD, but the relational literature based on the European population is sparse. Conclusions from these studies have been varied and, at times conflicting. For instance, a study conducted on Italian rheumatological patients concluded that RA patients had a higher risk for CD (Caio et al., 2018), which was consistent with our findings. However, other studies yielded conflicting results regarding the effect pattern (Francis et al., 2002; Moghtaderi et al., 2016). In the reverse relationship, our MR estimates contradict the available observational study, which suggested that the prevalence of autoimmune-related comorbidities (including RA) was more than three times higher among CD patients compared with a representative sample of the general Danish population (Grode et al., 2018). Furthermore, several other studies have not clarified a specific relationship between both diseases but rather explained a relationship of coexistence because of sharing a similar pathogenic mechanism and potential triggers, having a common genetic predisposition and a possible symptomatic overlap (Lerner and Matthias, 2015; Warjri et al., 2015; Therrien et al., 2020). Nevertheless, our MR study does not support a bidirectional causality between RA and CD. One explanation could be that the previously observed association of CD with RA is coincidental or thwarted by unknown confounders.

The causal effect of RA on CD in our study is of great significance for the diagnosis and treatment of CD. The NICE guidelines recommend testing high-risk adults with celiac serology (Downey et al., 2015). Immunoglobulin-A anti-tissue transglutaminase (IgA-TTG) testing is the recommended first-line approach for the diagnosis of CD in adults unless IgA-TTG is weakly positive, under which circumstance endomysial antibodies (EMA) concentration should also be tested (Lebwohl et al., 2018). Since we found that RA is a cause of CD, we recommend that patients with RA should be included as the high-risk population of CD and emphasize the significance of RA in the updated guidelines. Our research contributes to the existing body of knowledge about RA and CD, and the finding has substantial implications for public health, as it will anticipate the occurrence of CD in RA patients and give prevention and treatment measures for CD in RA patients. For example, surveillance examinations for RA patients should include not only regular rheumatological laboratory tests such as erythrocyte sedimentation rate (ESR) or concentrations of C-reactive protein (CRP) but also IgA-TTG testing and/or duodenal biopsy. In addition, the diet inflammatory potential has been demonstrated positively correlated with the risk of RA and increasing the probability of the risk of disease *via* superimposing effects with other risk factors (Xiang et al., 2022). So we also suggest RA patients adhere to a gluten-free diet (GFD), an anti-inflammatory diet, which is not only beneficial to prevent the development of CD but has also proven to reduce arthritic pain perception, control inflammation and improve the quality of life in RA patients (Guagnano et al., 2021).

There are several advantages of this research. First, the MR study design minimizes the residual confounding and reverse causality inherent in observational and epidemiological studies. Second, the genetic instruments explained 3% and 6.9% of the variation of RA and CD, with minimum *F* statistics of 210 and 205 respectively, consistent with the absence of weak instrument bias. Third, the MR analysis, IVW in particular, is precise enough to detect causal effects when all the IVs are valid, and produce consistent estimates using different MR techniques. Last, we provide evidence intensely supporting the causality of RA on CD from a genetic standpoint, the bidirectional analysis guaranteed the causality inference between RA and CD in both directions. Nevertheless, the limitations of the current study need to be considered. First, the RA and CD GWAS data were derived from patients of European ancestry, which may partially bias the outcomes. Applying the conclusions to populations of other ethnicities requires caution. Second, as the demographic data of all the GWAS participants are unavailable, the current study did not perform a gender-specific MR analysis although RA and CD are more prevalent in women than in men (Grode et al., 2018; Safiri et al., 2019). Third, there were likely overlapping involvers in the exposure and outcome research, but it is challenging to appraise the degree of sample overlap. Reassuringly, the strong IVs (*F* statistic much greater than 10) used in the study could minimize potential bias on sample overlap (Pierce and Burgess, 2013).

The results of this study demonstrated a causal association between genetically predicted RA on CD but did not indicate a causal effect of CD on RA. It is challenging to diagnose CD in patients with RA since their symptoms overlap in some ways. We need to keep in mind that patients with RA can have latent CD, in particular those with gastrointestinal symptoms. At the same time, we should not ignore symptoms of non-intestinal for CD and extra-articular manifestations for RA, like chronic fatigue, osteoporosis and anemia, which are important factors contributing to poor life quality for both RA and CD patients. After all, controlling disease activity, improving quality of life and enhancing subjective well-being is more important than curing the primary disease for patients with lifelong chronic diseases. In summary, the symptoms and indicators of CD need to be considered during diagnosing and managing any RA patients. Monitoring the intestinal mucosal events related to articular and extra-articular etiological pathways of RA may reduce the risk of CD in RA patients. GFD is a beneficial treatment and prevention measure that should be considered in RA and CD patients. Subsequent further studies or MR analysis based on updated and more extensive GWAS data are warranted to verify the mentioned results and elucidate the possible underlying mechanism.

## Data Availability

The original contributions presented in the study are included in the article/Supplementary Material, further inquiries can be directed to the corresponding authors.
