# Supplementary material for: Causal association between rheumatoid arthritis and celiac disease: A bidirectional two-sample mendelian randomization study
Source: Front Genet. 2022 Oct 18;13:976579. doi: 10.3389/fgene.2022.976579 (PMC9623103; doi:10.3389/fgene.2022.976579)
Supplement: Supplementary file 1 [file DataSheet1.docx]

Supplementary Material

## Supplementary Figures


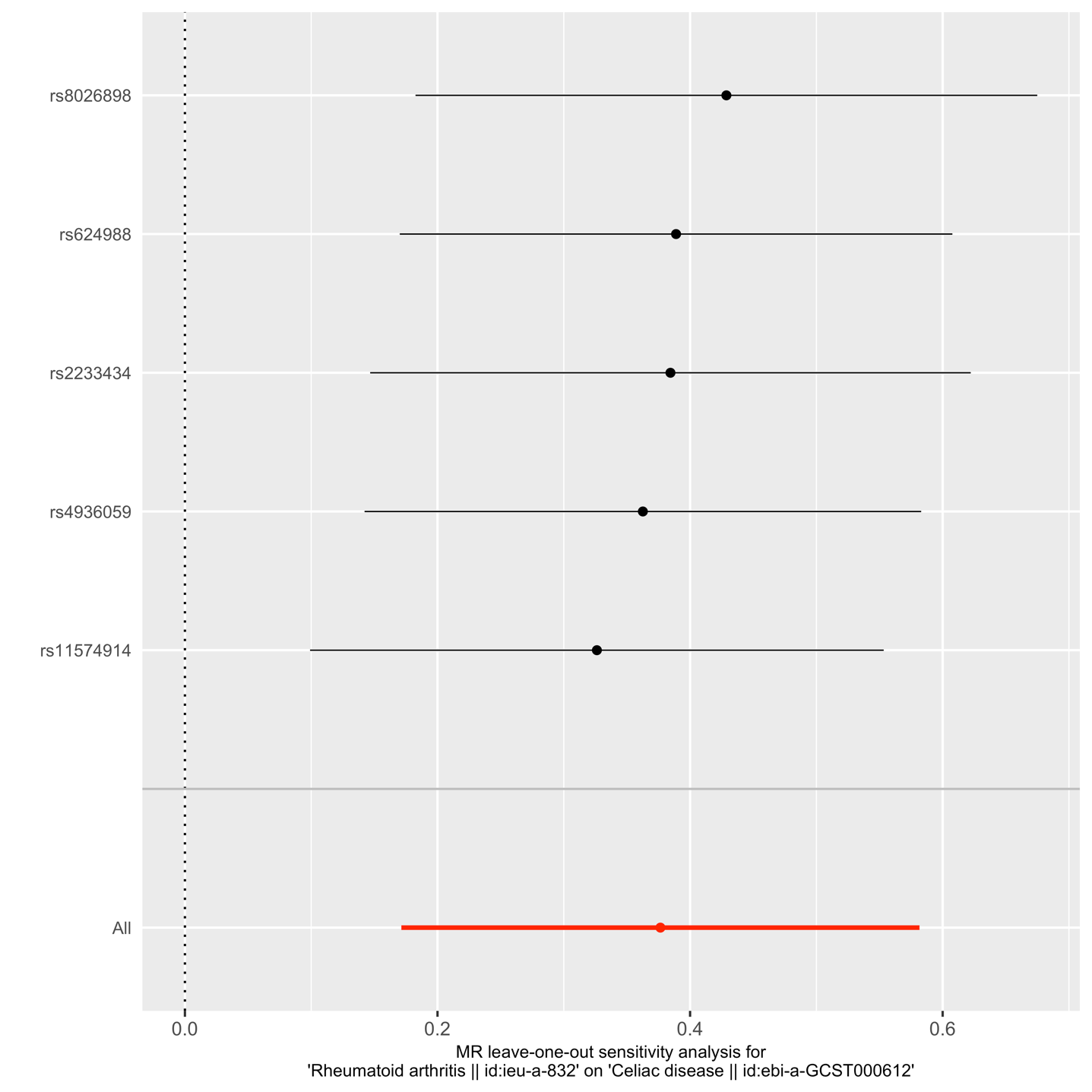


**Supplementary Figure 1.** The leave-one-out plot for the causal relationship of RA on CD.


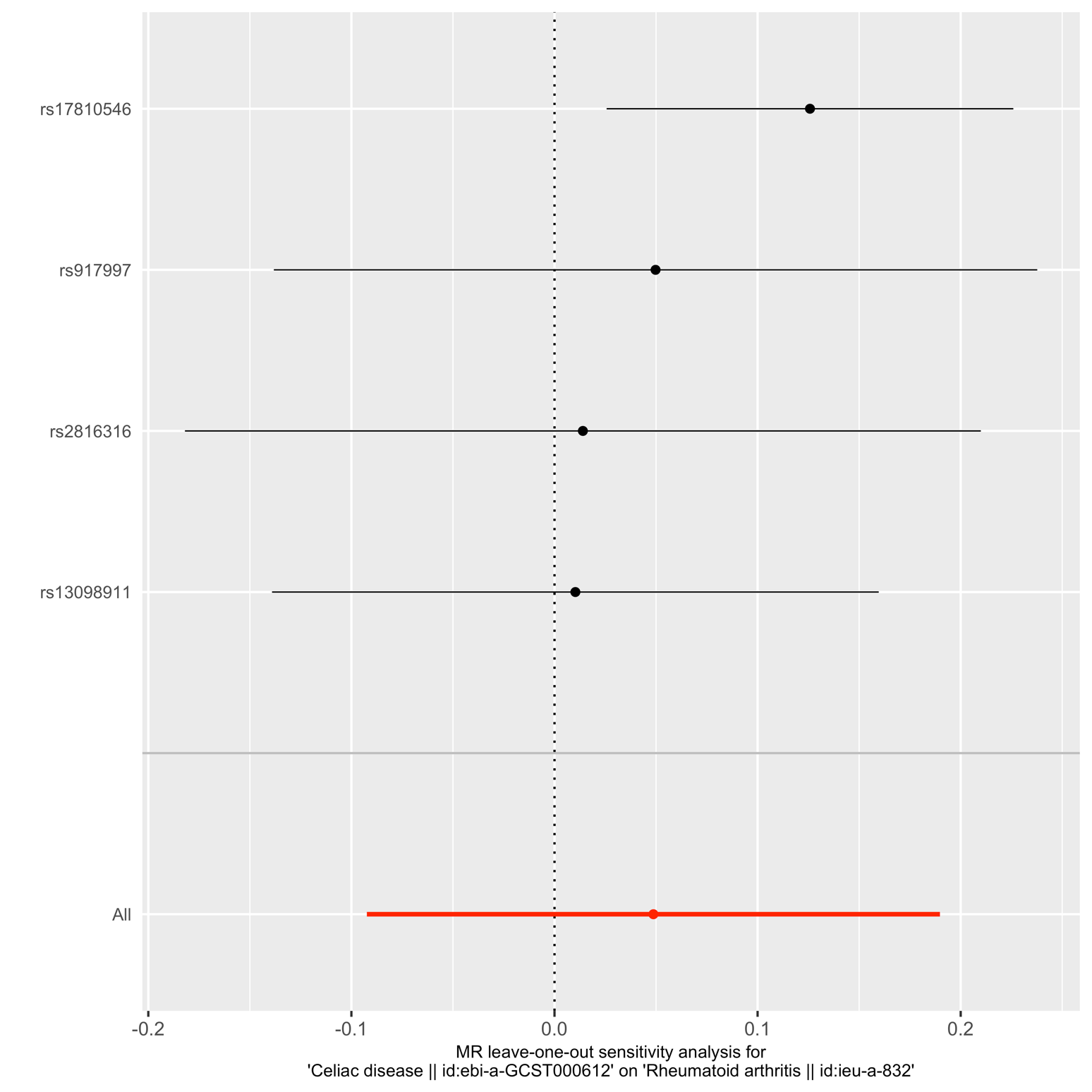


**Supplementary Figure 2.** The leave-one-out plot for the causal relationship of CD on RA.

## Supplementary Tables

| **Supplementary Table S1. Detailed information of studies and datasets used in the present study.** | | | | | |
| --- | --- | --- | --- | --- | --- |
| Exposure or outcome | PMID | Ancestry | Participants | GWAS ID | Web source |
| Rheumatoid arthritis | 24390342 | European | 14,361 cases and 43,923 controls | ieu-a-832 | https://gwas.mrcieu.ac.uk/datasets/ieu-a-832/ |
| Celiac disease | 20190752 | European | 4,533 cases and 10,750 controls | ebi-a-GCST000612 | https://gwas.mrcieu.ac.uk/datasets/ebi-a-GCST000612/ |
| Abbreviation: GWAS, Genome-wide association study. | | | | | |

| **Supplementary Table S2. Detailed information of SNPs used in the MR analysis of RA on CD.** | | | | | | | | | | | | | | |
| --- | --- | --- | --- | --- | --- | --- | --- | --- | --- | --- | --- | --- | --- | --- |
| SNP | Chr:BP | Nearest gene | EA/OA | MAF | Exposure (RA) | | |  | Outcome (CD) | | | *R^2^* | *F*-statistic ^1^ |  |
|  |  |  |  |  | OR | 95% CI | *P*-value |  | OR | 95% CI | *P*-value |  |  |  |
| rs11574914 | 9:34710338 | *CCL21* | A/G | 0.315 | 1.130 | 1.071-1.189 | 1.50E-13 |  | 1.076 | 1.017-1.135 | 0.014 | 0.0064 | 378.20 |  |
| rs2233434 | 6:44232920 | *NFKBIE, POLR1C* | G/A | 0.050 | 1.334 | 1.216-1.451 | 3.30E-08 |  | 1.107 | 0.990-1.225 | 0.088 | 0.0078 | 459.21 |  |
| rs4936059 | 11:128502496 | *RP11-744N12.3* | G/A | 0.340 | 1.099 | 1.046-1.151 | 4.60E-08 |  | 1.045 | 0.992-1.098 | 0.103 | 0.0040 | 233.59 |  |
| rs624988 | 1:117263790 | *GAPDHP64* | C/T | 0.412 | 0.918 | 0.867-0.969 | 4.60E-08 |  | 0.977 | 0.926-1.028 | 0.341 | 0.0036 | 210.40 |  |
| rs8026898 | 15:69991417 | *PCAT29* | A/G | 0.285 | 1.160 | 1.105-1.214 | 2.40E-17 |  | 1.039 | 0.984-1.094 | 0.173 | 0.0090 | 528.32 |  |
| Abbreviation: SNPs, single nucleotide polymorphisms; MR, Mendelian randomization; RA, rheumatoid arthritis; CD, celiac disease; Chr:BP, chromosome: base-pair position (GRCh37); EA/OA, effect allele/other allele; MAF, minor allele frequency; OR, odds ratio; 95% CI, 95% confidence interval. | | | | | | | | | | | | | | |
| ^1^ *F*-statistic were calculated using the following formula: *R^2^*(N-2)/(1-*R^2^*), where *R^2^* is the proportion of variance in RA explained by each instrument and N is the sample size of the GWAS for the SNP-RA association. | | | | | | | | | | | | | | |

| **Supplementary Table S3. Detailed information of SNPs used in the MR analysis of CD on RA.** | | | | | | | | | | | | | |
| --- | --- | --- | --- | --- | --- | --- | --- | --- | --- | --- | --- | --- | --- |
| SNP | Chr:BP | Nearest gene | EA/OA | MAF | Exposure (CD) | | |  | Outcome (RA) | | | *R^2^* | *F*-statistic ^1^ |
|  |  |  |  |  | OR | 95% CI | *P*-value |  | OR | 95% CI | *P*-value |  |  |
| rs13098911 | 3:46235201 | *CCR3* | T/C | 0.094 | 1.320 | 1.238-1.403 | 2.53E-11 |  | 1.060 | 1.007-1.113 | 0.030 | 0.0133 | 205.20 |
| rs17810546 | 3:159665050 | *IL12A-AS1* | G/A | 0.094 | 1.383 | 1.310-1.455 | 4.56E-18 |  | 0.962 | 0.913-1.011 | 0.110 | 0.0179 | 279.28 |
| rs2816316 | 1:192536813 | *LOC105371664* | A/C | 0.183 | 1.289 | 1.219-1.360 | 1.45E-12 |  | 1.030 | 0.995-1.066 | 0.110 | 0.0193 | 301.37 |
| rs917997 | 2:103070568 | *IL18RAP* | C/T | 0.221 | 0.793 | 0.734-0.852 | 5.97E-15 |  | 0.990 | 0.941-1.039 | 0.690 | 0.0185 | 288.02 |
| Abbreviation: SNPs, single nucleotide polymorphisms; MR, Mendelian randomization; CD, celiac disease; RA, rheumatoid arthritis; Chr:BP, chromosome: base-pair position (GRCh37); EA/OA, effect allele/other allele; MAF, minor allele frequency; OR, odds ratio; 95% CI, 95% confidence interval. | | | | | | | | | | | | | |
| ^1^ *F*-statistic were calculated using the following formula: *R^2^*(N-2)/(1-*R^2^*), where *R^2^* is the proportion of variance in CD explained by each instrument and N is the sample size of the GWAS for the SNP-CD association. | | | | | | | | | | | | | |
